# Isocyanate-functionalised graphene oxide and poly(vinyl alcohol) nacre-mimetic inspired freestanding films

**DOI:** 10.1039/d1na00792k

**Published:** 2021-11-12

**Authors:** Andrew J. Smith, Łukasz Figiel, Chaoying Wan, Tony McNally

**Affiliations:** International Institute for Nanocomposites Manufacturing (IINM), WMG, University of Warwick Coventry CV4 7AL UK t.mcnally@warwick.ac.uk

## Abstract

Nacre mimetic films based on 2-ureido-4[1*H*]-pyrimidinone (UPy) functionalised graphene oxide (GO) and poly(vinyl alcohol) (PVA) were readily prepared by self-assembly using a vacuum filtration method. The isocyanate (UPy) functionalisation of the PVA was confirmed from a combination of Fourier transform infrared spectroscopy (FTIR) and changes in *d*-spacing from X-ray diffraction (XRD) measurements and, of the GO by solid-state NMR measurements reported by the authors previously. This is the first example of nacre mimetic structures where both the nanoplatelet (GO) and polymer (PVA) components are functionalised with complimentary groups. The resulting films displayed substantial increases in Young's modulus (*E*) of 392% (GO1/PVA1), ultimate tensile strength (UTS, *σ*) of 535% (GO1/PVA1), elongation at break (*ε*_max_) of 598% (GO10/PVA5) and tensile toughness (*U*_T_) of 1789% (GO1/PVA10) compared to the un-functionalised GO analogues. The binding of UPy to both the GO and the PVA provides multiple routes by which these freestanding nacre mimetic films can dissipate applied loads.

## Abstract 2

Nacre mimetic films based on 2-ureido-4[1*H*]-pyrimidinone (UPy) functionalised graphene oxide (GO) and poly(vinyl alcohol) (PVA) were readily prepared by self-assembly using a vacuum filtration method.

## Graphical abstract

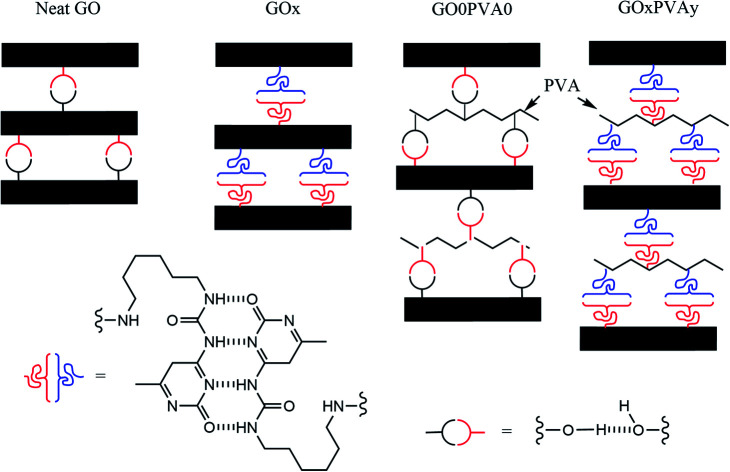

## 1. Introduction

Nacre, otherwise known as ‘mother-of-pearl’ is a remarkable material that displays mechanical properties that far exceed the sum of its component parts.^1^ Found in the shells of the mollusc family, nacre is composed of 95% by volume aragonite (the planar polymorph of CaCO_3_) and 5% biopolymer. The high nanoplatelet content results in a ‘brick and mortar’ structure whereby the aragonite platelets are arranged in a layered morphology and the biopolymer penetrates between these layers and behaves as a form of adhesive. Because of the highly structured morphology, cooperative energy dissipation mechanisms are present that efficiently relax localised stress over a large area, thus enabling absorption of large amounts of energy prior to failure.

Interest in nacre-mimetic materials has grown substantially in the past decades, in part due to the mechanical properties observed, but also the simplicity of manufacture and potential for production of multifunctional materials. It is widely understood that nacre-mimetic materials can be produced by vacuum-assisted filtration,^2–4^ solvent casting,^5–7^ and spraying,^8–10^ amongst others and there are examples where thermo-chromic transitions,^11^ enhanced thermal^12–14^ and electrical^15,16^ conductivity, and self-healing properties^17^ have been reported. It is widely recognised that cooperative effects between the hard (nanoplatelet) and soft (polymer) phase are key to maximising mechanical properties by introducing competing energy dissipation mechanisms.^18,19^ Thus, it is important to investigate techniques to increase the interfacial bonding between the two phases.

Previous literature has reported the introduction of both covalent^2,5,20^ and non-covalent^21–24^ bonding between the nanoplatelet and polymer components. Typically, graphene oxide (GO) is used as the nanoplatelet phase due to its reactive surface chemistry that allows for simple functionalisation *via* multiple methods.^25–27^ Notably, there are few examples of the introduction of non-covalent bonding to increase interactions. Park *et al.* reported the use of Mg^2+^ and Ca^2+^ ions to introduce ionic interactions between adjacent GO sheets however, the authors did not examine any effect on polymer-containing films.^21^ Critically, only minimal increases to Young's modulus (*E*) and tensile strength (*σ*) were achieved. More recently, Wang *et al.* functionalised GO with dopamine that was subsequently polymerised. Further treatment with 2-ureido-4[1*H*]-pyrimidinone (UPy) enabled grafting of the UPy onto the poly(dopamine) (PDA) and facilitated strong hydrogen bonding interactions between the PDA-capped GO sheets.^22^ Ultimately, this resulted in a 3.6 times increase in *σ* and a 10 times increase in tensile toughness (*U*_T_) when compare to a neat GO control.

In our previous work, we reported the successful functionalisation of GO with UPy using a simple reaction between the isocyanate of UPy and the multiple oxygenated surface groups present on GO (GOx).^23^ This facilitated increased hydrogen bonding between GO sheets and achieved increases in *E* (↑323%), *σ* (↑470%), *ε* (↑214%) and *U*_T_ (↑1117%). Critically, there was no polymer component in these materials and so the mechanical properties were not optimal. It was hypothesised that a polymer component could be functionalised using the UPy group to generate strong hydrogen bonding dimers with the GO component (also functionalised with UPy) that would greatly increase the interfacial interactions and ultimately achieve substantial increases in *E*, *σ*, *ε*_max_ and *U*_T_.

In this work, we report the functionalisation of poly(vinyl alcohol) (PVA) with UPy *via* a simple process. The UPy-functionalised PVA (PVAy) was then dissolved into DMSO before being mixed with a GOx dispersion and filtered under vacuum to produce nacre-mimetic films. To the best of our knowledge, this work represents the first example of nacre mimetic structures where both the nanoplatelet (GO) and polymer (PVA) components are functionalised with complimentary groups. It is also the first example of a nacre mimetic film of GOx containing a polymer component. The reaction of UPy and PVA is confirmed by Fourier-transform infrared (FTIR) spectroscopy. The morphology, chemical and mechanical properties of the GOx/PVAy nacre mimetic films were analysed by scanning-electron microscopy (SEM), X-ray diffraction (XRD) and quasi-static tensile testing, respectively.

## 2. Experimental

### 2.1 Materials

GO was purchased from Abalonyx, Norway in powder form and used with no further purification. 2-Amino-4-hydroxy-6-methylpyrimidine and DMF were purchased from Fisher Scientific and, hexamethylene diisocyanate (HDI) was purchased from VWR International. Dichloromethane (DCM), DMSO, isopropyl alcohol (IPA), *n*-pentane and poly(vinyl alcohol) (PVA), with a molecular weight of 89–98k and hydrolysed content of >99%, were purchased from Merck. All chemicals were used as received. Hydrophilic poly(tetrafluoroethylene) PTFE membranes were purchased from Merck. Molecular sieves (3 Å) were purchased from Fischer and activated by heating at 350 °C for at least 4 hours prior to use.

### 2.2 Synthesis methods

#### 2.2.1 Synthesis of 2-ureido-4[1*H*]-pyrimidinone (UPy)

UPy was synthesised as outlined in our previous work.^23^ Simply, 2-amino-4-hydroxy-6-methylpyrimidine (4.38 g, 35.0 mmol) was combined with an excess of HDI (38.0 cm^3^, 237 mmol), and heated at 100 °C for 24 hours. A white precipitate formed and was filtered under vacuum. The resulting solid was washed multiple times with *n*-pentane achieving a white powder in excellent yield (10.17 g, 34.7 mmol, 99%).

#### 2.2.2 Reaction of UPy with GO (GOx)

Functionalisation of GO with UPy was achieved using the method outlined in our previous work,^23^ previously reported by Stankovich *et al.*^28^ Typically GO (≈1.5 g) was combined in solid form with UPy at a weight ratio of GO : UPy = 1 : 1, 1 : 0.1 and 1 : 0.01 and will be referred to as GO50, GO10 and GO1, respectively (unmodified GO will be referred to as GO0 in this context). Anhydrous DMF (75 cm^3^) was added and the mixture degassed with N_2_ for 1 hour, then stirred for 24 hours. The resultant dispersion was coagulated in DCM (≈200 cm^3^) filtered under vacuum and washed with DCM. The brown powder obtained was dried overnight at 40 °C under vacuum.

#### 2.2.3 Reaction of UPy with PVA (PVAy)

PVA (9.96 g) was dissolved in DMSO (100 cm^3^) by heating at 60 °C until full dissolution was observed. The resulting mixture was then stored over activated molecular sieves (3 Å) for 24 hours to ensure anhydrous conditions. The dried PVA solution (10 cm^3^) was combined with UPy at a weight ratio of PVA : UPy = 1 : 0.1, 1 : 0.05 and 1 : 0.01 and will be referred to as PVA10, PVA5 and PVA1 from hereon (unmodified PVA will be referred to as PVA0 in this context). The mixture was degassed under a flow of Ar for 15 minutes. The mixture was then heated at 80 °C (whereby the UPy would dissolve) for 16 hours. Viscous products were achieved so DMSO (10 cm^3^) was added and the mixture agitated to aid dissolution of the polymer product. Precipitation was achieved in IPA (200 cm^3^) yielding a ‘cotton-like’ product. UPy loadings of 20 wt% and above could not be re-solubilised following the reaction so could not be isolated.

#### 2.2.4 Production of GOx/PVAy films

PVAy solutions of 1 wt% were achieved by dissolving PVAy (150 mg) in DMSO (15 cm^3^) at 80 °C until complete dissolution was observed. GOx powders (≈500 mg) were suspended in DMSO (50 cm^3^) and stirred for 2 hours. The PVAy solution (≈2.5 g) was then added dropwise under stirring. The mixture was stirred for 1 hour before being filtered through a hydrophilic PTFE membrane. The GOx/PVAy film and PTFE membrane were removed from the filtration setup and dried in a fan-assisted oven at 40 °C for 24 hours. The film could then be peeled from the PTFE membrane.

#### 2.2.5 Characterisation

FTIR measurements were made using a Bruker Tensor 27 spectrometer equipped with an attenuated total reflectance (ATR) crystal. Spectra were processed using OPUS analysis software between 500 and 4000 cm^−1^. A resolution of 2 cm^−1^ was used with an average of 12 scans used to acquire each spectrum.

Scanning Electron Microscopy (SEM) micrographs were obtained using a Zeiss Sigma instrument using an InLens detector at 10 kV. The samples imaged were sputter coated using an Au/Pd target prior to imaging.

XRD measurements were performed on a 3^rd^ generation Malvern Panalytical Empyrean instrument equipped with multicore (iCore/dCore) optics and a Pixcel3D detector operating in 1D scanning mode. A Cu tube was utilised giving Cu K_α1/2_ radiation (1.5419 Å) and a beam knife to reduce air scatter at the low angles. Scans were recorded in the range 4° to 30° 2*θ* with a step size of 0.0263° and a counting time of ∼130 s per step. Using the measured diffraction angle, 2*θ*, the interlayer spacing (*d*) was calculated using Bragg's law, *nλ* = 2*d* sin *θ* (where *λ* = 1.541 Å).

Quasi-static tensile testing was completed using an Instron 5800R machine equipped with a 500N load cell and an extension rate controlled at 1 mm min^−1^. Test specimens were cut into bars of width 10 mm and length 40 mm using a razor blade (thicknesses were determined from cross-sectional SEM analysis and the average taken from 10 measurements). The room temperature (RT) tensile mechanical properties were determined from an average of at least five (5) specimens. The Young's modulus was calculated from the gradient of the linear region of the stress–strain curve and tensile toughness calculated from the area under the stress–strain curves.

## 3. Results and discussion

**Scheme 1:**
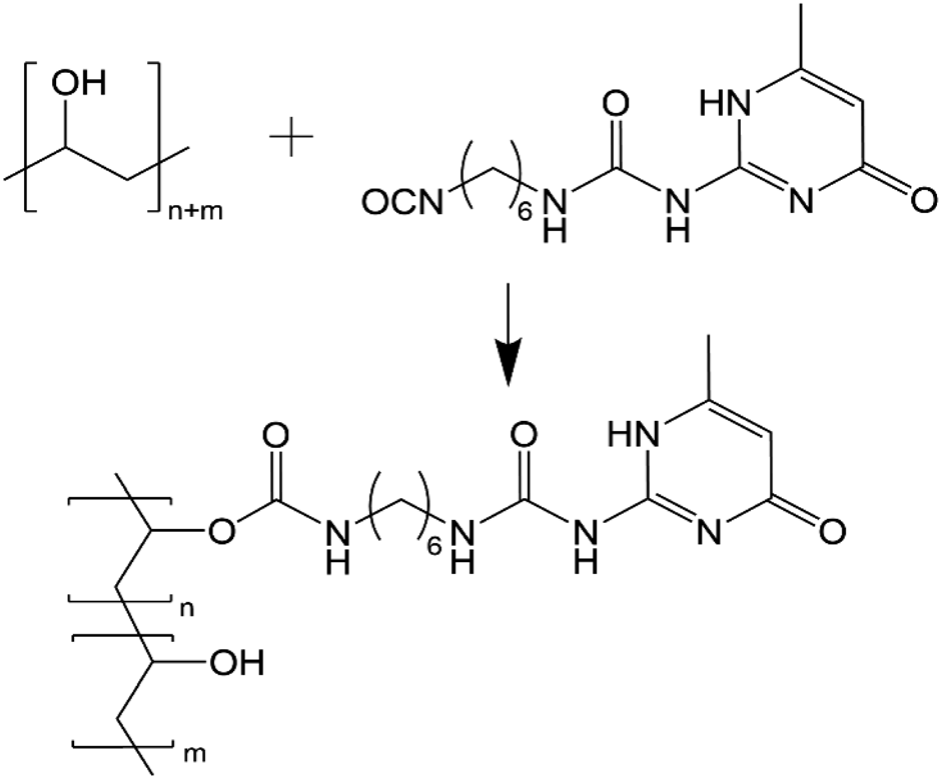
Reaction of PVA with UPy to produce the PVAy species.

Successful functionalisation of GO with the UPy group (GOx) has been described in our previous work.^23^ Of key importance from that work was that solid-state nuclear magnetic resonance (SSNMR) confirmed UPy reacted readily with the hydroxyl groups on the GO surface. PVA has abundant hydroxyl groups in the polymer chain and so grafting of UPy can be achieved easily (Scheme 1).

Isocyanates are reactive towards water, producing an amine and carbon dioxide. As a result, water could not be used in the reaction medium and anhydrous conditions were required. Dimethyl sulfoxide (DMSO) was found to effectively disperse GOx in our previous work and it was observed that PVA and UPy were soluble in DMSO when heated to 60 °C. Functionalisation of PVA was achieved by stirring an anhydrous mixture of PVA in DMSO with the UPy powder at room temperature for 24 hours. At high UPy content (*i.e.* >10 wt%), the mixture gelled due to the high degree of hydrogen bonding dimerization of the UPy pendant groups. As a result, these products could not be isolated.

**Fig. 1:**
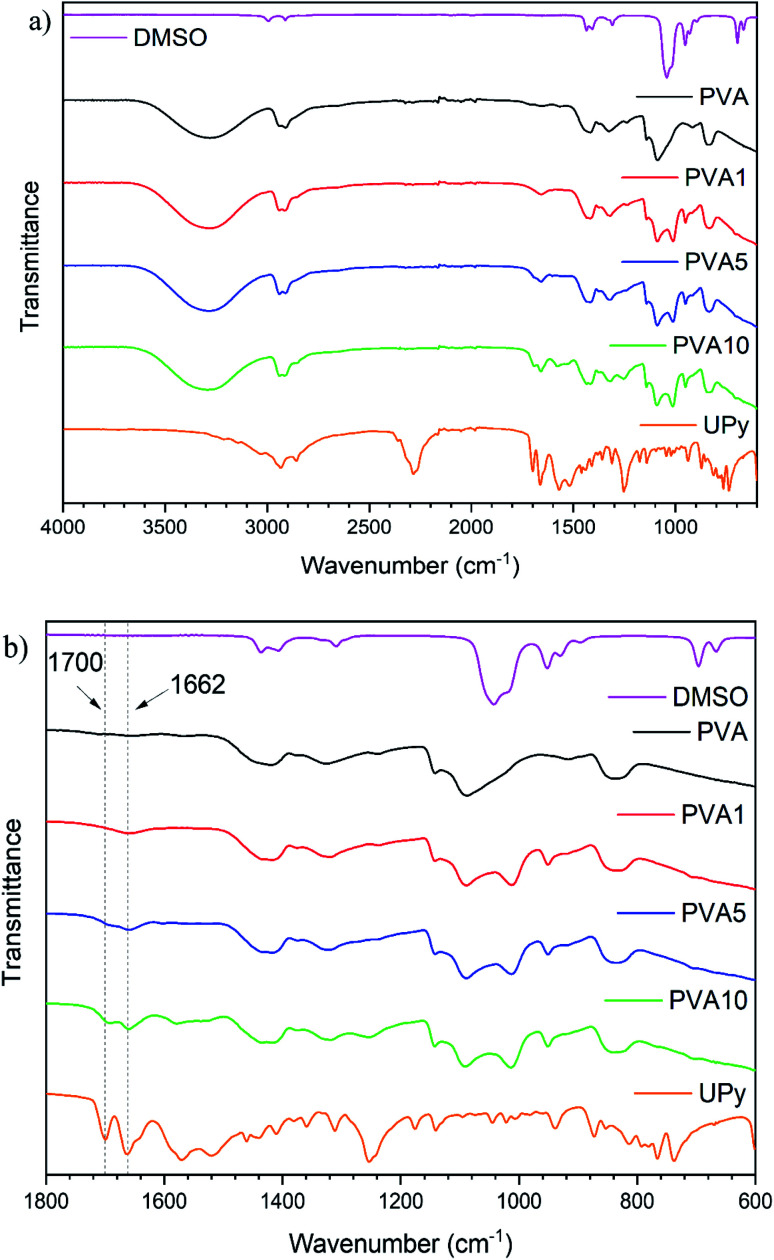
FTIR spectra of DMSO, UPy, PVA and functionalised PVAy where (a) full spectra from 4000 cm^−1^ to 600 cm^−1^ and (b) enlarged spectra from 1800 cm^−1^ to 600 cm^−1^. Diagnostic peaks are highlighted with a dashed line and the corresponding wavenumber.

FTIR spectroscopy was utilised to confirm successful functionalisation of PVA (Fig. 1a). Evolution of new peaks in the PVA spectrum are observed following the reaction with UPy, see Fig. 1b. Peaks at 1700 cm^−1^ and 1662 cm^−1^, corresponding to C=O stretches of the cyclic ketone and amide carbonyl of UPy, respectively, are detected in PVA1, PVA5 and PVA10 with increasing intensity at higher UPy content. Other peaks show an increase in intensity (*e.g.* 1253 cm^−1^ or 1013 cm^−1^) however, are much less prominent and/or overlap with solvent signals and so cannot be used to confirm a successful reaction. It is important to note there is no isocyanate peak at 2288 cm^−1^ in the PVAy samples. This confirms the terminal isocyanate of the UPy group has reacted with the PVA chain. The presence of the peaks at 1700 cm^−1^ and 1662 cm^−1^, and the absence of an isocyanate peak at 2288 cm^−1^, confirm the successful functionalisation of PVA with the UPy group.

**Fig. 2:**
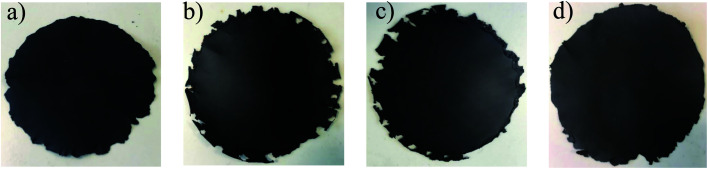
Digital photographs of representative GOx/PVAy films where (a) GO0/PVA10, (b) GO1/PVA10, (c) GO10/PVA10 and (d) GO50/PVA10. Sample diameter ∼100 mm.

Nacre-mimetic films were produced using a simple vacuum-assisted filtration approach. All GOx/PVAy films were produced at a 95 : 5 by weight ratio of GOx : PVAy and are named according to the UPy content on each component. For example, GO10/PVA5 contains 95 wt% of GO10 (10 wt% UPy content on GO) and 5 wt% PVA5 (5 wt% UPy content on PVA). Initially, GOx was dispersed in DMSO *via* stirring. A DMSO–PVAy solution was added before the mixture was stirred further. The resulting dispersion was filtered under vacuum and dried overnight. This allowed the nacre-mimetic film to be peeled from the substrate yielding freestanding films, see Fig. 2.

**Fig. 3:**
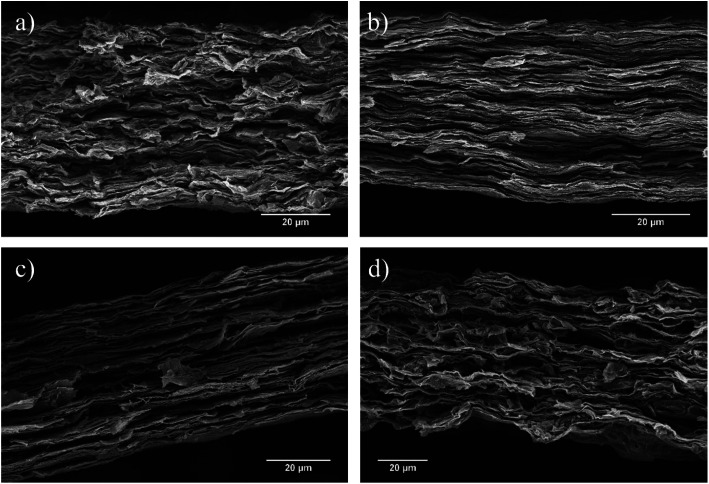
SEM micrographs of GOx/PVAy films where (a) GO0/PVA5, (b) GO1/PVA5, (c) GO10/PVA5 and (d) GO50/PVA5.

SEM was used to observe the cross-sectional layered structure of the nacre-mimetic films (Fig. 3). Distinct morphologies are present at different UPy content on GOx. The polymer-free films (GO0) have a defined layered morphology however, the cross-sectional face shows a more uniform morphology at the GO1 film. As the GOx nanoplatelets are functionalised with the UPy group, they are able to hydrogen bond to the PVAy component that aids in the construction of a defined morphology. At the GO10 content, the internal layers become highly ordered due to the increase in UPy dimers between the nanoplatelet and polymer components. Ultimately, the high UPy content in the GO50 films results in the agglomeration of the GOx and disrupts interactions with the PVAy component. As a result, the layered morphology is disrupted and the structure becomes less defined, see Fig. 3d.

**Fig. 4:**
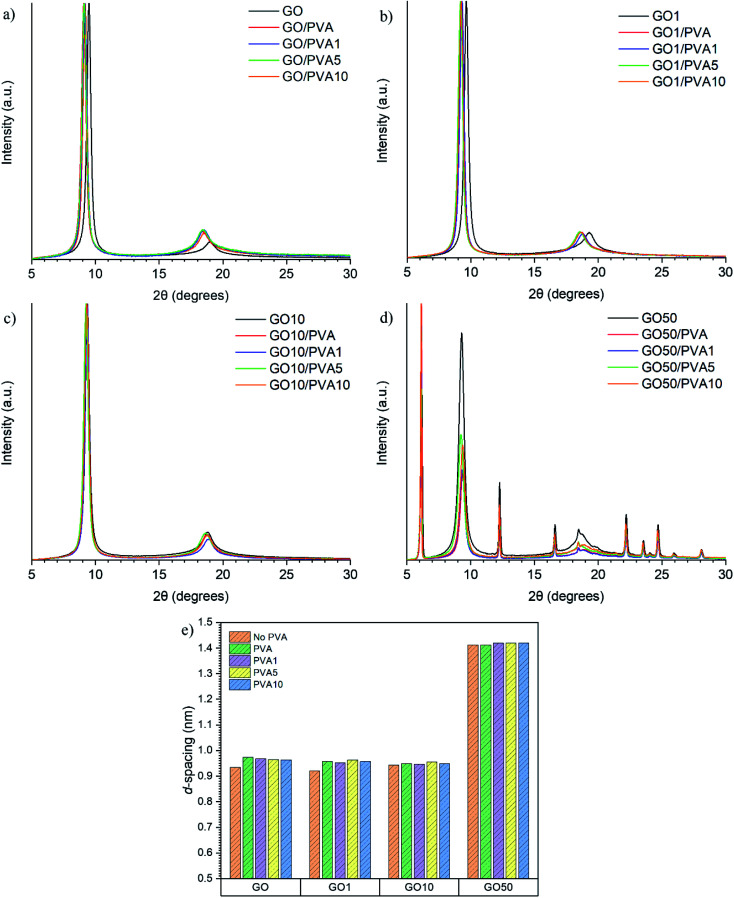
XRD diffractograms (a–d) and (e) the calculated *d* spacings of GOx/PVAy nacre-mimetic films.

Using the measured diffraction angle, 2*θ*, the interlayer spacing (*d*) of a crystalline material can be calculated using Bragg's law, *nλ* = 2*d* sin *θ* (where *λ* = 1.541 Å). The XRD patterns and calculated *d*-spacings are shown in Fig. 4. The *d*-spacing of the GO0/PVAy, GO1/PVAy and GO10/PVAy films increases when the PVA component is added. This is to be expected as the PVA penetrates between the GO layers and forces them to separate but, the slight increase is much less than would be expected given the size of the PVA chains. Perhaps the PVA aids the assembly of a more homogeneous layered structure through hydrogen bonding to the GO, possible tethering to the OH groups on the edge of the GO platelets. However, it is not until the ratio of GO to UPy is 1 : 1, *i.e.* (GO50), 100 times more UPy than for GO1, that there is a significant increase in *d*-spacing up to ∼1.419 for PVA10GO50. The XRD diffractogram shows the evolution of several new peaks, including a major intense peak at lower 2*θ*, ∼6° due to increased spacing (exfoliation) between the GO platelets and resulting in different layered morphology, see Fig. 3d.

**Fig. 5:**
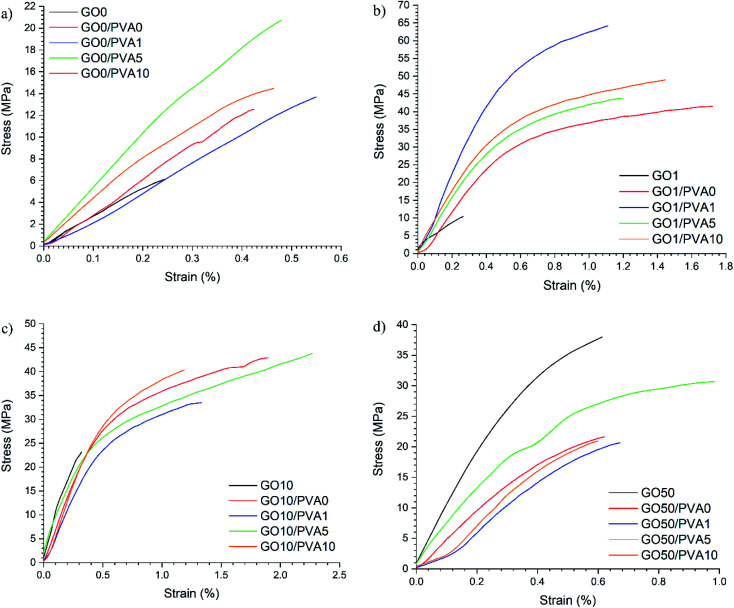
Representative stress–strain curves for the GOx/PVAy films where (a) GO0, (b) GO1, (c) GO10 and (d) GO50 based films (the moduli are determined from the steepest part of the slope of the stress–strain curves, within the linear elastic region).

Quasi-static tensile testing was completed to determine the mechanical properties of the films and representative stress–strain curves are shown in Fig. 5. It is interesting to note that the GO1/PVAy films had the highest *σ* = 62.85 ± 7.27 MPa, whilst GO10/PVAy films had the highest *ε*_max_ of up to 2.39 ± 0.43%. This highlights the complexity of the competing energy dissipation mechanisms occurring within the films.

**Fig. 6:**
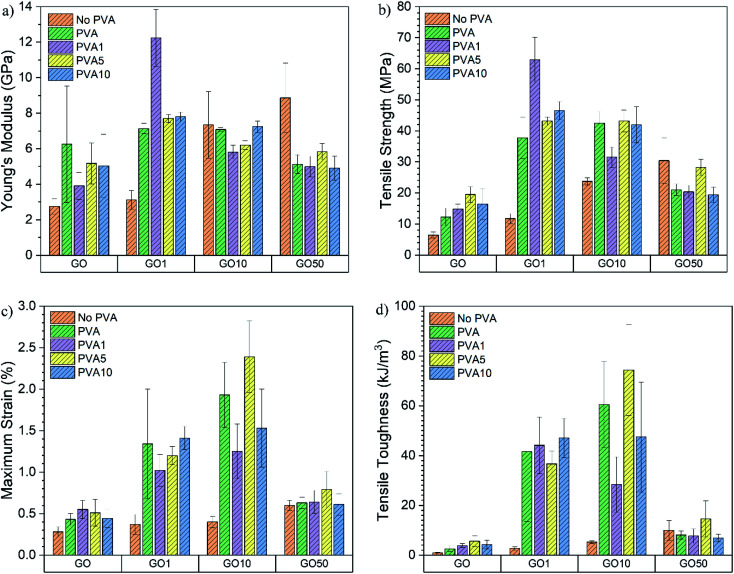
Comparison of (a) Young's modulus, (b) maximum tensile strength, (c) maximum strain and (d) tensile toughness of the GOx/PVAy films.

The mechanical properties calculated from the stress–strain curves are displayed as bar charts in Fig. 6. An increase in *E* from 2.75 ± 0.44 GPa for GO0 to 6.25 ± 3.28 GPa for GO0/PVA0 is obtained however, the error is relatively large. *E* increases for GO0/PVA1 (3.91 ± 0.76 GPa) compared to GO0 as the polymer enables a more efficient shear stress transfer across a layered GO-polymer structure,^29^ and suggests effective penetration of the PVA0 between the GO0 layers. For GO0/PVA5 and GO0/PVA10, *E* = 5.17 ± 1.15 GPa and 5.04 ± 1.78 GPa, respectively, confirming an increase in *E* compared to GO0/PVA1. This trend is supported by the XRD data which confirmed that increasing the *y* content of PVAy (*i.e.* from PVA1 to PVA5 to PVA10) results in a reduction in *d*-spacing. Reduced *d*-spacing suggests improved packing and thus stiffening by PVAy dominates and *E* increases.

There was a significant increase in *E* for GO1 (3.12 ± 0.53 GPa) upon addition of PVA0 to 7.14 ± 0.29 GPa for GO1/PVA0, an increase of 229%. For GO1/PVA1 there was a further increase in *E* to 12.24 ± 1.60 GPa, representing an increase of 392% and 171% compared to GO1 and GO1/PVA0, respectively. The UPy group (present on the GO1 and PVA1) forms strong interactions between the GO1 and PVA1 components, creating a 3D network of hydrogen bonding that increases the modulus by the PVAy. *E* is reduced for GO1/PVA5 and GO1/PVA10, down to 7.70 ± 0.24 GPa and 7.80 ± 0.26 GPa, respectively. The increasing *y* content on the PVAy results in a larger steric volume (UPy is much larger than hydroxyl pendant groups) of the PVAy component. This forces the GO1 nanoplatelets to separate and greater PVAy motion occurs. Separation of GO1/PVA5 and GO1/PVA10 is observed through the increased interlayer distance from XRD (GO1/PVA5 = 0.963 nm, GO1/PVA10 = 0.957 nm compared to GO1/PVA1 = 0.952 nm). However, the decrease in *d* is small, so other processes, such as tethering of the –OH groups on the GO platelet edges by PVA may also play a role in this behaviour.

**Fig. 7:**
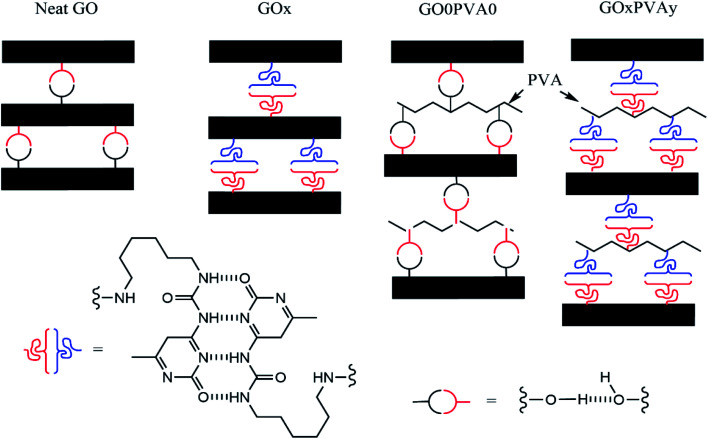
Schematic representation (not to scale) of the localisation of UPy functionalised PVA between GO layers, for *x* = 50.

The *E* of GO10 films ranges from 5.81 ± 0.39 GPa (GO10/PVA1) to 7.35 ± 1.88 GPa (GO10). The weight ratio of GOx : PVAy in these films = 95 : 5. As a result, increasing UPy content (*x*) in GOx (*i.e.* from GO1 to GO10) has a greater impact on the quantity of UPy groups in the structure compared to an increase to *y* in the PVAy (*i.e.* PVA1 to PVA10). It is therefore possible that in the GO10 films, the impact of changing the *y* content on the PVAy is not large enough to significantly affect *E*. The same is observed in the GO50 films where for all the GO50/PVAy films *E* is between 4.91 ± 0.67 GPa (GO50/PVA10) and 5.84 ± 0.45 GPa (GO50/PVA5). Interestingly, for GO50, *E* = 8.87 ± 1.95 GPa, significantly higher than the GO50/PVAy films. It is likely the PVAy disrupts the strong hydrogen bonding between the GO50 nanoplatelets and thus reduces collaborative stiffening of the film. The schematic diagram shown in Fig. 7 provides a physical interpretation of the positioning of the UPy functionalised PVA between the GO layers.

In the case of GO0, *σ* increases from 6.45 ± 0.97 MPa for GO0 to 12.29 ± 2.80 MPa for GO0/PVA0, an increase of 191%. *σ* = 14.78 ± 1.59 MPa for GO0/PVA1, 19.50 ± 2.52 MPa for GO0/PVA5 and 16.45 ± 5.00 MPa for GO0/PVA10. Critically, a significant increase in *σ* of 302% is observed between GO0 and GO0/PVA5. The GO0 nanoplatelets assemble into a porous structure.^30^ The pores localise stress and disrupt the dissipation of energy through the film. Most probably coalescence of pores into a major defect also causes a decrease in UTS. The PVAy occupies the pores within the film and bridges the GOx nanoplatelets. As a result, energy is dissipated over a larger volume as the number of pores/defects is reduced with the addition of PVAy, resulting in an increase in *σ*. For the GO1 film *σ* = 11.74 ± 1.62 MPa however, addition of PVA0 yields an increase of 321% to 37.74 ± 6.75 MPa (GO1/PVA0). For GO1/PVA1 *σ* = 62.85 ± 7.27 MPa, corresponding to a substantial increase of 535% over GO1. The UPy dimers between the GO1 and PVA1 create strong interfacial interactions that greatly increase the energy required to break the 3D network. The UPy content in GO1/PVA1 is optimal to maximise *σ* where the hydrogen bonding is most cooperative. GO1/PVA5 and GO1/PVA10 have *σ* = 43.16 ± 1.31 MPa and 46.55 ± 2.89 MPa, respectively, most likely due to an increase in hydrogen bonding between PVAy chains hindering cooperative interactions with the GO1. Despite this, *σ* still increases by close to 400% for both the GO1/PVA5 and GO1/PVA10 films when compared to GO1.

An increase in *σ* is recorded from 23.75 ± 1.13 MPa for GO10 to 42.50 ± 3.80 MPa for GO10/PVA0. No further increase in *σ* is observed for GO10/PVA1 (31.55 ± 3.26 MPa), GO10/PVA5 (43.17 ± 3.52 MPa) or GO10/PVA10 (41.95 ± 5.79 MPa) however, all have increased *σ* when compared to GO10, most likely due to increased plastic deformation. It is clear that increasing *y* content on the PVAy (*i.e.* PVA0 to PVA1, PVA5 and PVA10) does not increase *σ*. This suggests that hydrogen bonding between adjacent GO10 sheets dominates hydrogen bonding between the GO10 and PVAy. Finally, *σ* decreases from 30.43 ± 7.37 MPa for GO50 to 21.03 ± 1.81 MPa, 20.39 ± 2.11 MPa, 28.27 ± 2.63 MPa and 19.41 ± 2.52 MPa for GO50/PVA0, GO50/PVA1, GO50/PVA5 and GO50/PVA10, respectively. GO50 has higher *σ* when compared to GO0, GO1 and GO10, suggesting the strongest hydrogen bonding between GOx sheets is present in the GO50 film. It is likely the PVAy disrupts the hydrogen bonding between the GO50 sheets, thus weakening interfacial interactions. As a result, stress cannot be dissipated between adjacent GO50 sheets resulting in a decrease in *σ*.

GO0 had *ε*_max_ = 0.28 ± 0.06% with an increase of 154%, to 0.43 ± 0.07%, recorded for GO0/PVA0. PVA0 hydrogen bonds to GO0 through pendant hydroxyl groups and the interactions to the GO0 allow the GO0 sheets to slide over each other as the PVA0 chains disentangle. GO0/PVA1, GO0/PVA5 and GO0/PVA10 recorded *ε*_max_ of 0.55 ± 0.11%, 0.51 ± 0.16% and 0.44 ± 0.11% respectively, all showing an increase over the GO0 film. The increase in *ε*_max_ between GO0/PVA0 and GO0/PVA1, GO0/PVA5 and GO0/PVA10 is relatively small due to the lack of UPy functionalisation on the GO0 component.

For GO1/PVA0, *ε*_max_ = 1.34 ± 0.66%, a 362% increase over GO1 that measured 0.37 ± 0.12%. In this case, the UPy on the GO1 binds to the hydroxyl groups of the PVA0 and the flexibility of the PVA0 enables extension through disentangling of the polymer chain. Similarly, for GO10/PVA0, *ε*_max_ = 1.93 ± 0.39%, compared to 0.40 ± 0.07% for GO10. *ε*_max_ decreased for GO1/PVA1 (1.02 ± 0.19%) compared to GO1/PVA0 whilst GO10/PVA1 (1.25 ± 0.33%) decreases compared to GO10/PVA0. It is expected that *ε*_max_ would increase due to formation of UPy dimers between the GO1/GO10 and PVA1 components. The UPy six-carbon chain is longer than the hydroxyl groups present in PVA0 and so should extend further prior to failure.^23^ A decrease in *ε* suggests the UPy dimers between the GO1 and PVA1 create anchor points that prevent the PVA1 chains from disentangling. For GO10/PVA10, *ε*_max_ = 2.39 ± 0.43% that corresponds to a 598% increase over the GO10 film (0.40 ± 0.07%). This represents the optimal GOx/PVAy composition for *ε* and is due to the cooperative effect of elongation of UPy dimers and disentangling of the PVA10 chains. This allows the GO10 sheets to slide over each other enabling higher elongation. Critically, optimal UPy content is different for maximising *σ* or *ε*_max_.

Tensile toughness (*U*_T_) is a strong indicator of the overall ability of the films to absorb energy prior to failure. Calculated from the area under the stress–strain curve, *U*_T_ depends on *E*, *σ* and *ε*. For GO0, *U*_T_ = 0.89 ± 0.31 kJ m^−3^ that increases 281%, to 2.50 ± 1.19 kJ m^−3^, for GO0/PVA0. Subsequently, for GO0/PVA1, GO0/PVA5 and GO0/PVA10, *U*_T_ = 3.86 ± 0.79 kJ m^−3^, 5.61 ± 2.06 kJ m^−3^ and 4.24 ± 1.79 kJ m^−3^ respectively. PVAy aids in construction of a cooperative 3D network through occupation of pores within the GO0 structure. This enables dissipation of energy throughout the structure by eliminating localised stress points to increase *σ* and *ε*_max_, resulting in an increase in *U*_T_.

*U*_T_ = 2.63 ± 0.73 kJ m^−3^ for GO1 but critically, GO1/PVA10 yields a 1789% increase to 47.04 ± 7.81 kJ m^−3^. This highlights the impact of strong UPy dimers between the GO1 and PVA10. The increased interfacial bonding strength, extension of the UPy dimer and disentangling of the PVA10 chains enable GO1/PVA10 to absorb and dissipate energy through multiple competing mechanisms. Maximum *U*_T_ was 74.33 ± 18.26 kJ m^−3^ for GO10/PVA5, corresponding to a 1421% increase over GO10 and 6615% over GO0. GO10/PVA5 also had optimal *ε*_max_ and it is clear that the UPy content on the GO10 and PVA5 yields strong cooperative interactions, producing a 3D network that effectively dissipates energy due to applied quasi-static loads.

Similar to GO0, GO1, GO10 and GO50, *σ* and *U*_T_ of the freestanding GOx/PVAy films are lower than that reported in related literature with ionically^5,6^ and covalently^3,4^ cross-linked films reporting higher absolute values for *σ* and *U*_T_. Despite this, *E* and *ε*_max_ are comparable in some cases. There is no widely accepted standard for tensile testing of films of this nature. Consequently, the values for *E*, *σ*, *ε*_max_ and *U*_T_ can often be misleading. It is more valid to compare *E*, *σ*, *ε*_max_ and *U*_T_ to controls produced and tested using equivalent methodology as this provides a more realistic indication of the impact of any modifications. Functionalisation of GO and PVA with UPy for nacre-mimetic materials is proven to increase *E*, *σ*, *ε*_max_ and *U*_T_ significantly when compared to GO0, GO1 and GO10 controls. The increases to *E*, *σ*, *ε*_max_ and *U*_T_ (between GOx and GOx/PVAy films) eclipse those reported previously.^3,4,22^

## 4. Conclusions

In this work, it was shown that UPy reacts readily with PVA and GO at room temperature, as confirmed by FTIR. The resulting PVAy dissolves in GOx–DMSO dispersions and was found to self-assemble into a nacre-mimetic layered structure through vacuum-assisted filtration, thus producing the first GOx/PVAy films of their kind. The resulting films display substantial increases in *E*, *σ*, *ε*_max_ and *U*_T_ of 392% (GO1/PVA1), 535% (GO1/PVA1), 598% (GO10/PVA5) and 1789% (GO1/PVA10) compared to GOx analogues, respectively. Critically, the optimal UPy content for GOx and PVAy was different for *E* and *σ* (GO1/PVA1), *ε*_max_ and *U*_T_ (GO10/PVA5), confirming the energy dissipation mechanisms within the GOx/PVAy structures are complex and competing.

## Conflicts of interest

The authors report no conflicts of interest.

## Supplementary Material

## References

[cit1] Jackson A. P., Vincent J. F., Turner R. M. (1988). Proc. R. Soc. London, Ser. B.

[cit2] Gao Y., Liu L.-Q., Zu S.-Z., Peng K., Zhou D., Han B.-H., Zhang Z. (2011). ACS Nano.

[cit3] Wan S., Peng J., Li Y., Hu H., Jiang L., Cheng Q. (2015). ACS Nano.

[cit4] Wan S., Hu H., Peng J., Li Y., Fan Y., Jiang L., Cheng Q. (2016). Nanoscale.

[cit5] Cui W., Li M., Liu J., Wang B., Zhang C., Jiang L., Cheng Q. (2014). ACS Nano.

[cit6] Sarin S., Kolesnikova S., Postnova I., Ha C.-S., Shchipunov Y. (2016). RSC Adv..

[cit7] Ming S., Chen G., He J., Kuang Y., Liu Y., Tao R., Ning H., Zhu P., Liu Y., Fang Z. (2017). Langmuir.

[cit8] Dwivedi G., Flynn K., Resnick M., Sampath S., Gouldstone A. (2015). Adv. Mater..

[cit9] Bai H., Walsh F., Gludovatz B., Delattre B., Huang C., Chen Y., Tomsia A. P., Ritchie R. O. (2016). Adv. Mater..

[cit10] Pan X. F., Gao H. L., Lu Y., Wu C. Y., Wu Y. D., Wang X. Y., Pan Z. Q., Dong L., Song Y. H., Cong H. P., Yu S. H. (2018). Nat. Commun..

[cit11] Peng J., Cheng Y., Tomsia A. P., Jiang L., Cheng Q. (2017). ACS Appl. Mater. Interfaces.

[cit12] Zeng X., Ye L., Yu S., Li H., Sun R., Xu J., Wong C. P. (2015). Nanoscale.

[cit13] Yao Y., Zeng X., Wang F., Sun R., Xu J.-b., Wong C.-P. (2016). Chem. Mater..

[cit14] Shen Z., Feng J. (2018). ACS Appl. Nano Mater..

[cit15] Li Y. Q., Yu T., Yang T. Y., Zheng L. X., Liao K. (2012). Adv. Mater..

[cit16] Zhang M., Huang L., Chen J., Li C., Shi G. (2014). Adv. Mater..

[cit17] Zhu B., Jasinski N., Benitez A., Noack M., Park D., Goldmann A. S., Barner-Kowollik C., Walther A. (2015). Angew. Chem., Int. Ed..

[cit18] Smith B. L., Schaffer T. E., Viani M., Thompson J. B., Frederick N. A., Kindt J., Belcher A., Stucky G. D., Morse D. E., Hansma P. K. (1999). Nature.

[cit19] Li L., Ortiz C. (2014). Nat. Mater..

[cit20] Cheng Q., Wu M., Li M., Jiang L., Tang Z. (2013). Angew. Chem., Int. Ed..

[cit21] Park S., Lee K.-S., Bozoklu G., Cai W., Nguyen S. T., Ruoff R. S. (2008). ACS Nano.

[cit22] Wang Y., Li T., Ma P., Zhang S., Zhang H., Du M., Xie Y., Chen M., Dong W., Ming W. (2018). ACS Nano.

[cit23] Smith A. J., Kelly N. L., Figiel Ł., Wan C., Hanna J. V., Farris S., McNally T. (2020). ACS Appl. Nano Mater..

[cit24] Smith A., Wan C., Figiel Ł., Farris S., McNally T. (2020). Compos. Sci. Technol..

[cit25] Wu J., Huang G., Li H., Wu S., Liu Y., Zheng J. (2013). Polymer.

[cit26] Abbas S. S., Rees G. J., Kelly N. L., Dancer C. E. J., Hanna J. V., McNally T. (2018). Nanoscale.

[cit27] Song Y., Gao Y., Rong H., Wen H., Sha Y., Zhang H., Liu H.-J., Liu Q. (2018). Sustainable Energy Fuels.

[cit28] Stankovich S., Piner R. D., Nguyen S. T., Ruoff R. S. (2006). Carbon.

[cit29] Podsiadlo P., Kaushik A. K., Arruda E. M., Waas A. M., Shim B. S., Xu J., Nadivada H., Pumplin B. G., Lahann J., Ramamoorthy A., Kotov N. A. (2007). Science.

[cit30] Medina L., Nishiyama Y., Daicho K., Saito T., Yan M., Berglund L. A. (2019). Macromolecules.

